# The diagnostic dilemma of idiopathic intracranial hypertension in a child with acute lymphoblastic leukemia: COVID-19 or cytosine arabinoside?

**DOI:** 10.1186/s12883-022-02689-z

**Published:** 2022-05-02

**Authors:** Rim Rakez, Wiem Boufrikha, Sana Lakhal, Amel Boughammoura, Mohamed Adnene Laatiri

**Affiliations:** 1 Department of Hematology at Fattouma Bourguiba Hospital, Monastir, Tunisia; 2 Neurology at Ruspina Neurology Clinic, Monastir, Tunisia

**Keywords:** Acute lymphoblastic leukemia, COVID-19, Idiopathic intracranial hypertension, Headache, Case report

## Abstract

**Background:**

Idiopathic intracranial hypertension is a rare neurological condition among children. Its manifestations vary from mild headaches to loss of vision. Although rare, COVID-19 infection and high dose cytosine arabinoside have been reported as risk factors for this neurological disorder. In patients with acute leukemia, idiopathic intracranial hypertension diagnosis is simple, but finding its etiology can be difficult.

**Case presentation:**

We report a case of a 9-year-old boy with an ongoing treatment for T-acute lymphoblastic leukemia presenting with persistent headaches and diplopia. A diagnosis of idiopathic intracranial hypertension was retained based on clinical, imaging and laboratory findings. Due to its rarity, we describe its clinical and therapeutic features and highlight the challenging etiological dilemma between COVID-19 infection and high dose cytosine arabinoside administration.

**Conclusion:**

Persistent headache in a pediatric patient with leukemia can be due to many neurological disorders other than leukemic relapse. Given the improvement of the neurological symptoms after the SARS-CoV-2 PCR negativization and the successful re-introduction of high dose cytosine Arabinoside, the diagnosis of idiopathic intracranial hypertension associated with Covid-19 infection was withheld.

**Supplementary Information:**

The online version contains supplementary material available at 10.1186/s12883-022-02689-z.

## Background

Pseudotumor cerebri syndrome (PTCS), also known as idiopathic intracranial hypertension (IIH), is a rare condition [1]. The main symptom of this disorder is a persistent headache associated or not with signs of elevated intracranial pressure (ICP) such as nausea, vomiting, diplopia and visual obscurations [2]. In pediatric patients treated for acute leukemia, these symptoms are first considered by hematologists as a sign of leukemic relapse. Instead, in this situation, they revealed a diagnosis of IIH, which requires a prompt diagnosis and thorough evaluation and treatment. Numerous factors can lead to IIH including medications, immune disorders, and bacterial and viral infections [3].

We report the case of child with COVID-19 and pre-existing T-acute lymphoblastic leukemia (T-ALL) who was treated with high dose of cytosine arabinoside (HiDAC).

## Case report

A 9-year-old non obese male patient, with no medical history, was diagnosed with T-ALL in January 2021. He was included in the ‘Very High Risk’ (VHR) arm of the EORTC CLCG 58951 protocol with a complete remission post induction maintained until post interval therapy (Additional file 1).

During his hospitalization for the first block (R1), he received Dexamethasone 20 mg/m^2^/day (day 1 to 5), high dose methotrexate 5 g/m^2^/day (day1), an intrathecal injection with methotrexate, cytarabine and hydrocortisone (day 2), vincristin 1.5 mg/m^2^/day (day 1 and day 6), and HiDAC 4 g/m^2^/day (day 5) (Table 1).

**Table 1:** Timeline table of the case report

Dates	Relevant Past Medical History and Interventions		
January 2021	A 9-year-old non obese male patient, diagnosed with T-ALL, with no medical history.		
Dates	Summaries from Initial and Follow-up Visits	Diagnostic Testing (including dates)	Interventions
**August**	Intense holocranial headache, visual blurring and diplopia. Physical examination was normal.	1/RT-qPCR for SARS-CoV-2: positive 2/LP: normal CSP components, negative culture with normal cytological result.	Clinical monitoring
**2021**	Persistent symptoms	1/Ophthalmological examination: bilateral stage II papilledema and paralysis of the sixth right cranial nerve. 2/MRI and MRV: normal 3/LP: elevated opening pressure	Acetazolamide 125 mg per day
**August 2021**	Persistent diplopia, visual blurring and headache.	1/RT-qPCR for SARS-CoV-2: positive 2/LP: normotensive CSF	1/Acetazolamide 500 mg per day. 2/LP: A volume of 20 ml of CSF was taken.
**September 2021**	Improvement of the headache after LP but persistence of visual disturbances. Diplopia disappeared within a week after dose adjustment of treatment.	1/ RT-qPCR for SARS-CoV-2: negative 2/ Ophthalmological examination: persistent papilledema	Acetazolamide 500 mg per day

Since induction, he received 7 lumbar punctures (LP) containing 30 mg of cytarabine each and a cumulative dose of 13 g of intravenous HiDAC.

The R1 course was uneventful. Hepatic and renal tests were normal and the patient remained apyretic.

On day 14 of the cure, the patient became neutropenic and was diagnosed fortuitously with an asymptomatic COVID-19 infection contracted from his father who tested positive for this virus. Close monitoring for cardiorespiratory state, fever and complete blood cells count was started. Injections of granulocyte-colony stimulating factor were prescribed for 3 days until the normalization of neutrophil count. Given the stability of hemodynamic and respiratory status and the normality of lab findings we opted for outpatient treatment.

On day 21 of the cure (day 8 of the COVID-19 infection), he presented with intense holocranial headaches, visual blurring and diplopia. Neurological examination showed no focal deficit or ataxia and physical examination was normal. Neurological relapse was suspected and the patient underwent a LP. Cerebrospinal fluid (CSF) examination showed proteinorrachia at 10 mg/dL (normal level < 40 mg/dL), glycorrhachia at 61 mg/dL (glycorrhachia / glycemia ratio > 0.5), 7 red cells and 1 leukocyte (lymphocyte) without blasts on cytological analysis. Microbiological culture of CSF was negative, eliminating encephalitis and meningitis.

Ophthalmological examination found bilateral stage II papilledema as well as a paralysis of the sixth right cranial nerve.

Brain and orbital magnetic resonance imaging (MRI) showed no evidence of hemorrhage or cerebral mass and magnetic resonance venography (MRV) revealed no cerebral venous thrombosis. The patient was commenced on acetazolamide at a dose 125 mg per day with close neurological monitoring.

Control RT-qPCR for SARS-CoV-2, done on day 11 of the infection, remained positive and the patient was still complaining of diplopia, visual blurring and headache.

A second LP was performed on day 12 of the COVID-19 infection showing a jet CSF with an opening pressure at 350 mmH_2_O. A volume of 20 ml of CSF was taken inducing a partial alleviation of the headache but the visual disturbances persisted.

The diagnosis of IIH was confirmed and acetazolamide was prescribed at a dose of 500 mg per day. The headache was alleviated rapidly and diplopia disappeared within a week after treatment.

Control RT-qPCR for SARS-CoV-2 on day 20 of the infection was negative. An ophthalmological examination revealed a persistent papilledema.

Given the context of our patient, we concluded that this intracranial hypertension (ICH) was due to this virus and the patient was readmitted, 3 days after the negative control of the PCR, to receive the second block (R2) of VHR arm (Table 1).

LP was done on day 2 of the course with intrathecal injection of methotrexate and cytarabine, highlighting a normotensive CSF with normal cytological result. The R2 course went without incident and the patient remained apyretic. Twenty-eight days after the R2 cure, the patient was admitted to receive the third block (R3) of VHR arm including HiDAC. The course went with no neurological complaint (Table 1).

## Discussion and conclusion

PTCS is an uncommon neurological disease which comprises idiopathic and secondary causes of ICH. It is characterized by the presence of increased ICP with no evidence of a parenchymal mass, central nervous system infection or ventriculomegaly [4].

The incidence of pediatric IIH is slightly higher than that arising from secondary causes (0.63 and 0.32 per 100.000 respectively) [5] and it depends mainly on pubertal status. For post pubertal children, IIH is associated with the same adult risk factors for this disease including obesity and female sex, whereas for pre pubertal patients, IIH is not related to obesity and it affects girls and boys equally [4].

The symptoms of ICH are less evident in children and adolescents [6]. The most frequent sign at diagnosis is headache, described commonly as a pressure-like or throbbing frontal pain [2, 3]. Symptoms of raised ICP, like nausea and vomiting can be present, with or without ophthalmological manifestations like diplopia, blurring vision, transient visual obscurations and abducens nerve palsy [7]. Other signs, such as olfactory dysfunction or cognitive decline are more reported in adult patients [3, 8].

Regarding this constellation of symptoms, the diagnosis of IIH should always be suspected in a child with such neurological manifestations and a series of exams must be started urgently.

A thorough ophthalmologic examination is the first and the most important step to highlight ICH. In fact, the presence of papilledema at the time of diagnosis underlines a higher risk of vision loss [3, 9]. Neuroimaging techniques including MRI and MRV are then demanded to identify parenchymal lesions and to exclude cerebral venous thrombosis. The last diagnostic procedure is to measure the opening pressure of the CSF on LP [3].

Given the background of our patient whose symptoms were primarily suggestive of a T-ALL relapse in the central nervous system, an LP was performed first. This certainly allowed us to make a rapid diagnosis of the ICH by measuring CSF pressure, but it could have caused serious neurological complications such as cerebral herniation [10].

To retain the diagnosis of IIH, Friedman et al. revised its diagnostic criteria involving papilledema, normal neurologic examination (except for cranial nerve abnormalities), normal brain imaging with no abnormalities in venous status, normal CSF composition and elevated LP opening pressure (> 28 cmH2O in children) [11].

In our case, although the diagnosis was easy, the challenge was to find the etiology of this IIH. In fact, countless factors may cause pediatric ICH including drugs (tetracycline, cytarabine, corticosteroid withdrawal….). IIH following HiDAC is well-known to be rare [12]. It highlights the linear association between CSF concentrations and plasma levels of cytosine arabinoside [13]. Since our patient was only in his first line of T-ALL treatment, the cumulative dose of HiDAC received was insignificant to cause neurological toxicity. Hence, HiDAC was not considered as an IIH risk factor for our patient.

Regarding the importance of cytosine arabinoside in the treatment of T-ALL, we decided to continue the protocol. We initially introduced intrathecal cytarabine (30 mg) in R2 block to test the child’s tolerance for this drug then we continued HiDAC in block R3 with close neurological monitoring and both courses went without incident.

On the other hand, viral infections have been reported to cause ICH [14]. Since the outbreak of the COVID-19 pandemic, a lot of cases of IIH secondary to SARS-CoV-2 infection have been reported, revealed mainly by a persistent headache [9, 15]. This virus is well-known to have a potential neurotropism with several manifestations that occur in 45.5% of infected patients, such as headache, acute cerebrovascular disease and impaired consciousness [9, 16].

The first case of IIH associated with COVID-19 infection was reported in May 2020, in a 35-year-old female patient with no medical history [17]. The mechanism of IIH in COVID-19 is still controversial. One of the recent hypotheses indicates that it can be caused by high CSF neurofilament light chain associated with the increased CSF pressure. The other main mechanism related to IIH is venous thromboembolism which is frequently associated with a coagulation dysfunction in infected patients [9, 18].

To the best of our knowledge, our patient represents the first case of pediatric IIH associated with COVID-19 infection with a history of an ongoing treatment of T-ALL.

This etiological dilemma should not delay the onset of the treatment because of the risk of vision loss. According to The Idiopathic Intracranial Hypertension Trial Treatment, acetazolamide or topiramate is the front-line therapeutic approach [3, 19]. LP may be considered to drain CSF in order to decrease ICP [6]. Surgical intervention can be performed in case of intolerance or non-adherence to the first-line therapy [3].

Generally, headache is the first symptom to resolve within few weeks of the treatment, but papilledema could persist until 4 to 5 months [20]. In our case, after prescribing acetazolamide and a releasing LP, headache and diplopia improved rapidly within a week, whereas papilledema is still persistent 1 month after the treatment. This favorable evolution appearing after a negative control of the COVID-19 PCR and the successful re-introduction of cytosine arabinoside is a further argument in favor of IIH induced by COVID-19 infection.

Moreover, to establish whether IIH is due to COVID-19 or HiDAC is a challenge. In fact, for the case series published by Silva et al., IIH secondary to COVID-19 was more prevalent in women which is in agreement with the incidence of IIH in the general population. In our case, the patient is a child and as we mentioned previously that during pre- puberty, IIH affects boys and girls equally [4, 9]. For HiDAC associated IIH, few cases have been published and there no gender difference has been reported [12, 21]. For both etiologies, the most predominant symptom for IIH is headache and other neurological signs can appear over the time [9, 21]. According to the literature, the onset of symptoms in IIH secondary to medication can take weeks to months. However, in COVID-19, ICH can appear days to weeks after the presumed infection [9, 18, 22]. In our case, symptoms appeared 3 weeks after receiving HiDAC and 1 week after the positivity of the RT-qPCR for SARS-CoV-2, which makes the situation more difficult. Furthermore, treatment is the same for both COVID-19 and HiDAC -associated IIH, which consists in acetazolamide as the first -line therapy independently of the origin. Sings of immediate improvement can be shown in both cases but sometimes papilledema can take time to resolve [9, 18, 21]. In our case, the re-introduction of HiDAC is a supporting element that IIH is induced by COVID-19 infection.

Although IIH diagnosis in an ALL-pediatric patient is easy to establish, finding its etiology remains challenging in the era of the COVID-19 pandemic. Regardless of the cause, IIH is an emergency and the onset of treatment should, in no way, be delayed.

## Supplementary Information

**Additional file 1.** VHR arm treatment according to EORTC 58951 Protocol.

**Additional file 2.**

## Data Availability

The datasets referenced for this study are available from the corresponding author.

## Abbreviations

PTCS: Pseudotumor cerebri syndrome
IIH: Idiopathic intracranial hypertension
ICP: Intracranial pressure
T ALL: T acute lymphoblastic leukemia
HiDAC: High Dose Cytosine Arabinoside
VHR: Very High Risk
LP: Lumbar Puncture
CSF: Cerebrospinal fluid
MRI: Magnetic resonance imaging
MRV: Magnetic resonance venography
ICH: Intracranial hypertension
