# Supplementary figures and images for: The diagnostic dilemma of idiopathic intracranial hypertension in a child with acute lymphoblastic leukemia: COVID-19 or cytosine arabinoside?
Source: BMC Neurol. 2022 May 2;22:163. doi: 10.1186/s12883-022-02689-z (PMC9058734; doi:10.1186/s12883-022-02689-z)

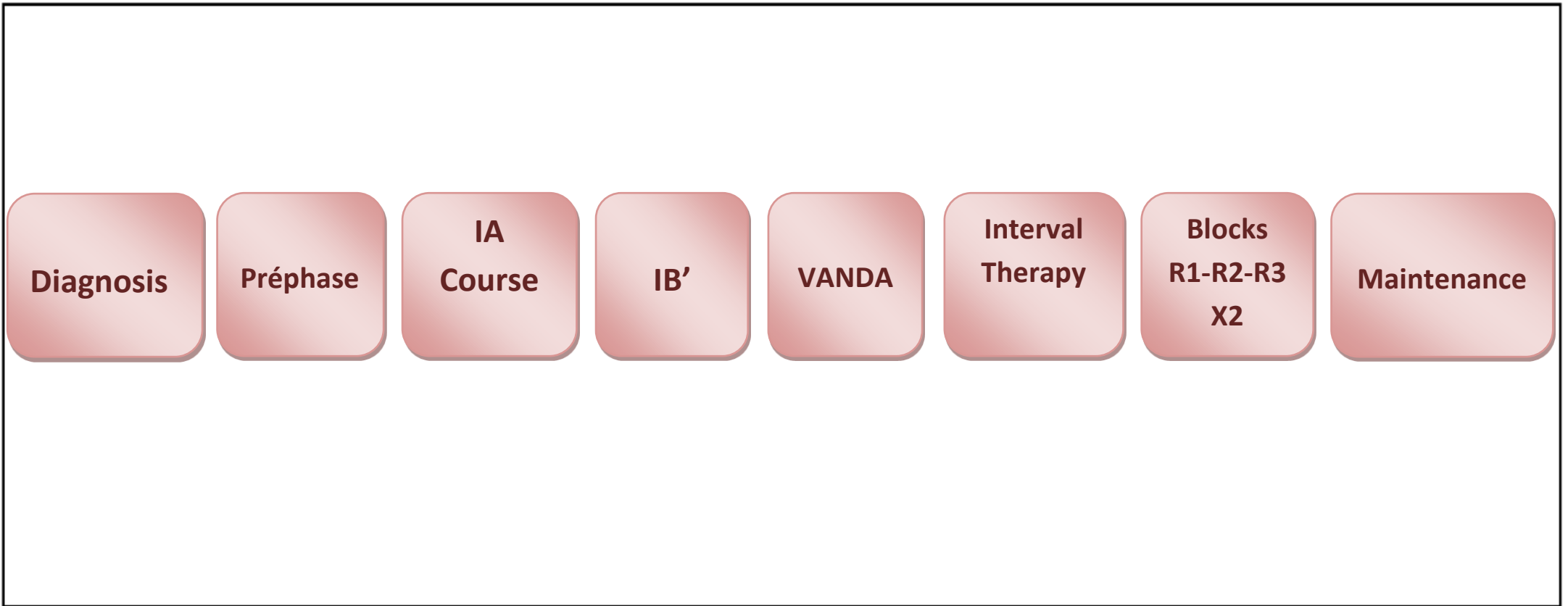

Annex 1: VHR arm treatment according to EORTC 58951 Protocol

Supplement: Supplementary file 1 — Additional file 1. VHR arm treatment according to EORTC 58951 Protocol. [file 12883_2022_2689_MOESM1_ESM.pdf]
