# Supplementary material for: The diagnostic dilemma of idiopathic intracranial hypertension in a child with acute lymphoblastic leukemia: COVID-19 or cytosine arabinoside?
Source: BMC Neurol. 2022 May 2;22:163. doi: 10.1186/s12883-022-02689-z (PMC9058734; doi:10.1186/s12883-022-02689-z)
Supplement: Supplementary file 2 — Additional file 2. [file 12883_2022_2689_MOESM2_ESM.pdf]

	Drug	Dose	Route	Number of administrations	Days
R1	DXM	10 mg/m^2/12h	p.o	10	1 to 5
	6-MP	100 mg/m^2	p.o	5	1 to 5
	VCR	1.5 mg/m^2	i.v	2	1 and 6
	HD-MTX	5 g/m^2	i.v	1	1(24h)
	Triple i.t chemo	-	i.t	1	2
	HiDAC	2 g/m^2/12h	i.v	2	5
	L-A'ase	25,000 UI/m^2	i.v	1	6
R2	DXM	10 mg/m^2/12h	p.o	10	1 to 5
	6-TG	100 mg/m^2	p.o	5	1 to 5
	VCR	1.5 mg/m^2	i.v	1	1
	HD-MTX	5 g/m^2	i.v	1	1(24h)
	Triple i.t chemo	-	i.t	1	2
	CPM	500 mg/m^2	i.v	2	3 and 4
	DNR	50 mg/m^2	i.v	1	5
	L-A'ase	25,000 UI/m^2	i.v	1	6
R3	DXM	10 mg/m^2/12h	p.o	10	1 to 5
	HiDAC	2 g/m^2/12h	i.v	4	1 and 2
	VP-16	150 mg/m^2	i.v	3	3,4 and 5
	Triple i.t chemo	-	i.t	1	2
	L-A'ase	25,000 UI/m^2	i.v	1	6

I.t chemo: intra-thecal chemotherapy, i.v: intravenous, P.o: per os, DXM: Dexamethasone, 6-MP: 6-Mercaptopurine, VCR: Vincristine, HD-MTX: High Dose Methotrexate, HiDAC: High Dose Cytosine Arabinoside, L-A'ase : L Asparaginase, 6-TG: 6-Thioguanine, CPM: Cyclophosphamide, DNR :Daunorubicine.

Blocks R1-R2-R3 of VHR arm according to EORTC 58951 protocol
